# Supplementary material for: Colorectal cancer progression to metastasis is associated with dynamic genome-wide biphasic 5-hydroxymethylcytosine accumulation
Source: BMC Biol. 2025 Apr 16;23:100. doi: 10.1186/s12915-025-02205-y (PMC12004686; doi:10.1186/s12915-025-02205-y)
Supplement: Supplementary file 1 — Additional file 1: Fig. S1 Total counts of hMeDIPseq peaks (enriched over input) show metastatic tumours to the liver have more 5hmC enrichment compared to primary tumours, and hMeDIPseq peak loci in metastatic liver tumours cluster towards the colon. Fig. S2 STRING and Gene Ontology analyses of 5hmC marked genes that have differential expression in metastasis. Fig. S3 A visual examination of the 5hmC data on an integrative genomics viewer (IGV), showing a pattern of 5hmC ‘recovering’ at sites where 5hmC was present in normal colon tissue, absent in the colon tumour and recovered in the metastatic tumours. Fig. S4 CRISPR-Cas9 strategy for TET1/TET2/TET3 triple knock out (TKO) in SW480 cells and effects on 5hmC accumulation. [file 12915_2025_2205_MOESM1_ESM.pdf]

S4)

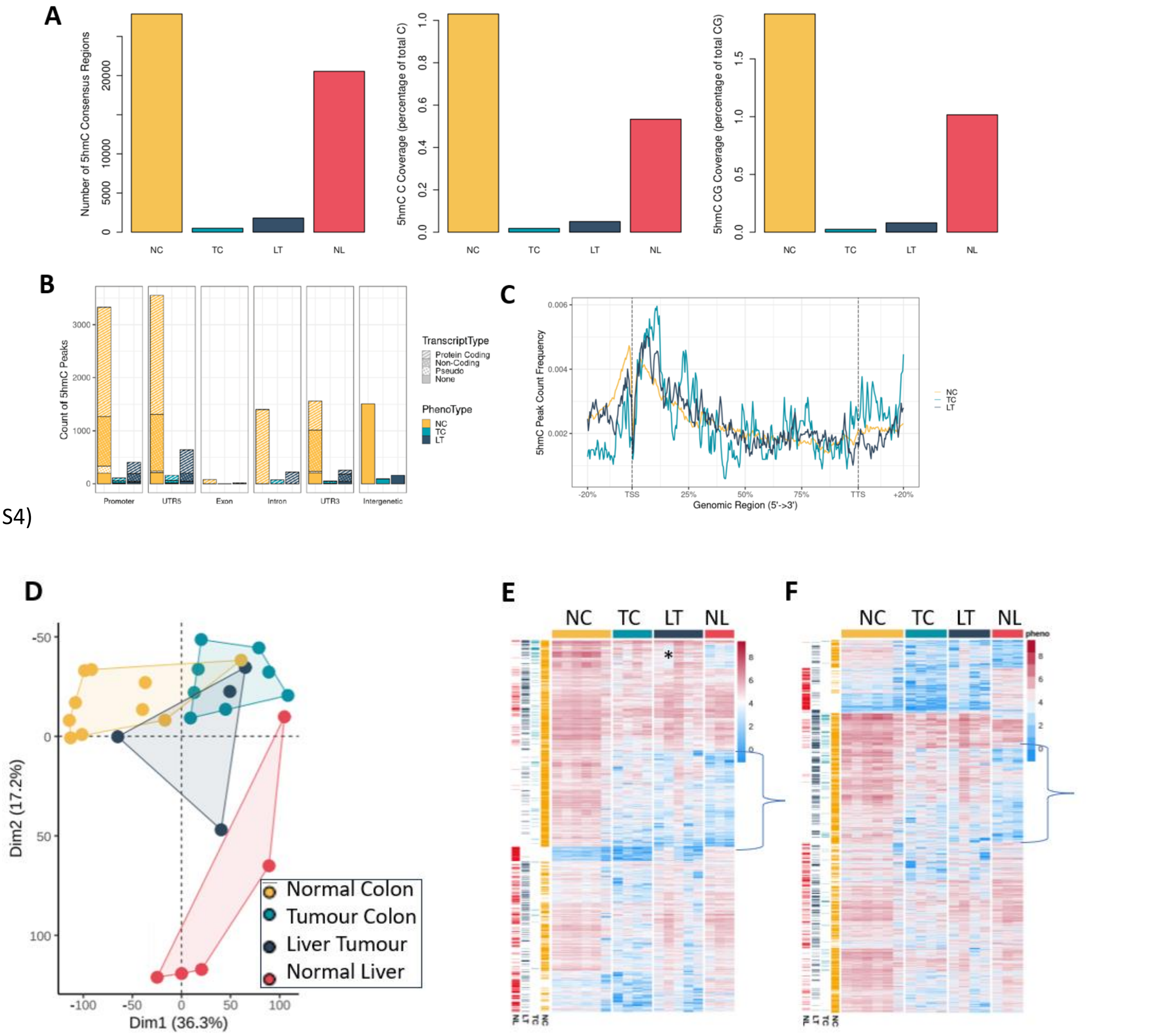

Figure S1: Total counts of hMeDIPseq peaks (enriched over input) shows metastatic tumours to the liver has more 5hmC enrichment compared to primary tumours, and hMeDIPseq peak loci in metastatic liver tumours cluster towards the colon A) Number of 5hmC consensus peaks, coverage as a percentage of total C and as a percentage of total CGs. Colour scheme: yellow is normal colon (NC), blue is primary Tumour in Colon (TC), dark blue is metastases to liver tumours (LT) and red is normal liver tissue (NL). **B)** Breakdown of features (Promoters, coding genes, noncoding genes, exons, introns, UTRs at 5hmC peaks **C**) 5hmC count frequency around transcriptional start site (TSS), gene body, and transcriptional termination sites (TSS), for NC (yellow), TC (blue), LT (dark blue). **D-F**) PCA and Heatmap analysis without the potentially contaminated liver tumour sample, mentioned in Fig 2. D) PCA analysis shows tighter clustering towards the colon samples when the outlier sample is removed. E) Heatmap analysis including the contaminated sample (*), F) Heatmap analysis without this sample. The brackets indicate a cluster of loci (Group 4 in Fig 2) within the metastasis sample that have 5hmC peaks in liver tumours similar to normal colon, and which don't have 5hmC in normal liver.

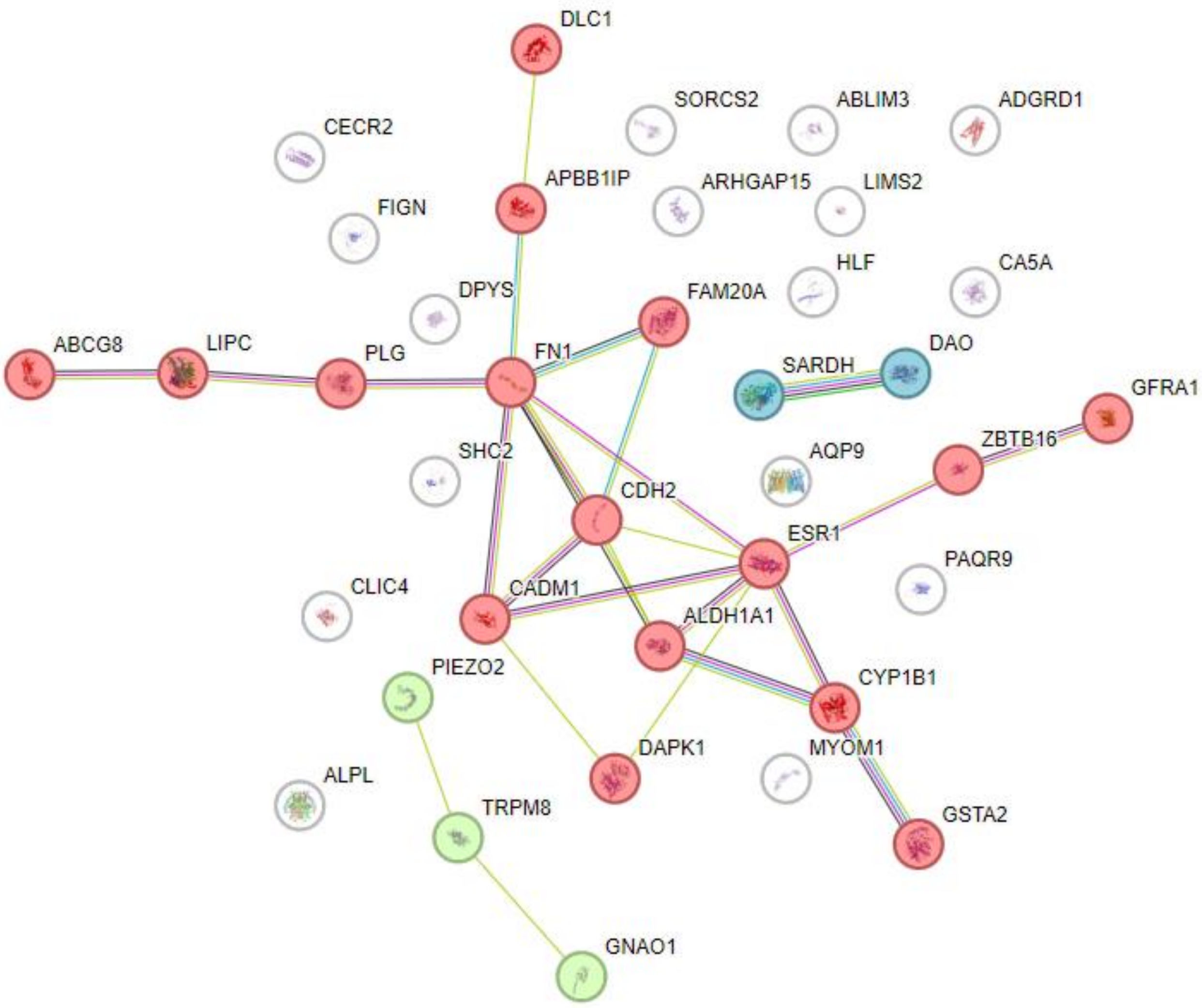

Biological Process (Gene Ontology)				
GO-term	description	count in network	strength	false discovery rate
GO:1904017	Cellular response to Thyroglobulin triiodothyronine	2 of 3	2.97	0.0021
GO:0007161	Calcium-independent cell-matrix adhesion	2 of 4	2.85	0.0028
GO:0033631	Cell-cell adhesion mediated by integrin	2 of 6	2.67	0.0043
GO:1904179	Positive regulation of adipose tissue development	2 of 8	2.55	0.0060
GO:0035357	Peroxisome proliferator activated receptor signaling pathway	2 of 9	2.5	0.0068
GO:0023035	CD40 signaling pathway	2 of 10	2.45	0.0078
GO:0051918	Negative regulation of fibrinolysis	2 of 13	2.34	0.0111
GO:0035313	Wound healing, spreading of epidermal cells	2 of 13	2.34	0.0111
GO:0030949	Positive regulation of vascular endothelial growth factor rec...	2 of 14	2.3	0.0123
GO:0010763	Positive regulation of fibroblast migration	2 of 16	2.25	0.0147
GO:2000811	Negative regulation of anoikis	2 of 19	2.17	0.0194
GO:0033627	Cell adhesion mediated by integrin	4 of 42	2.13	6.88e-05
GO:0035987	Endodermal cell differentiation	4 of 44	2.11	6.88e-05
GO:0034113	Heterotypic cell-cell adhesion	3 of 36	2.07	0.0014
GO:0071634	Regulation of transforming growth factor beta production	3 of 39	2.03	0.0016

Figure S2: STRING and Gene Ontology analyse of 5hmC marked genes that have differential expression in metastasis. Settings: Interactive sources; High confidence 0.700, 1st shell query proteins only; K-means clustering 15. Statistical Background (the whole genome). Full gene list: List: *ABCG8, , ABLIM3, ADGRD1, ALDH1A1, ALPL, APBB1IP, AQP9, ARHGAP15, CA5A, CADM1, CDH2, CECR2, CYP1B1, DAO, DAPK1, DPYS, ESR1, FAM20A, FIGN, FN1, GFRA1, GNAO1, GSTA2, HLF, LINC01348, LIPC, LOC105373215, LOC124904475, MIR99AHG, MYOM1, PAQR9, PIEZO2, PLG, SARDH, SHC2, TRPM8, ZBTB16, LIMS2, DLC1, CLIC4, GNAI, SORCS2, IL6-AS1*, Gene Ontology output with STRING. Table – Gene ontology output of above STRING

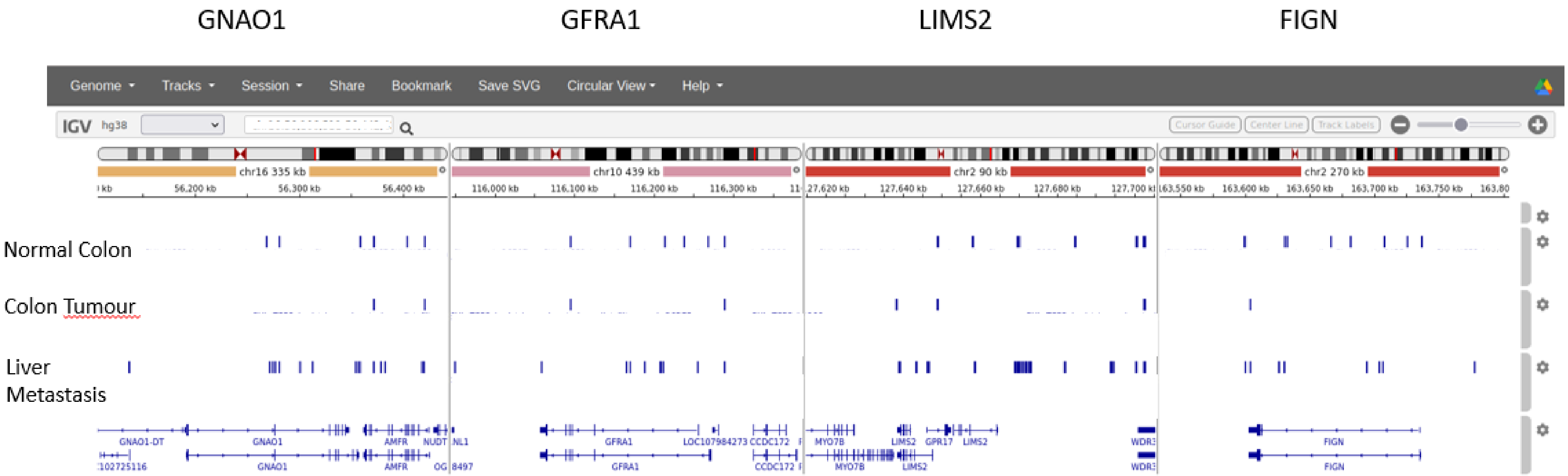

Figure S3 A visual examination of the 5hmC data on an integrative genomics viewer (IGV), showing a pattern of 5hmC “recovering” at sites where 5hmC was present in normal colon tissue, absent in the colon tumour, and recovered in the metastatic tumours.

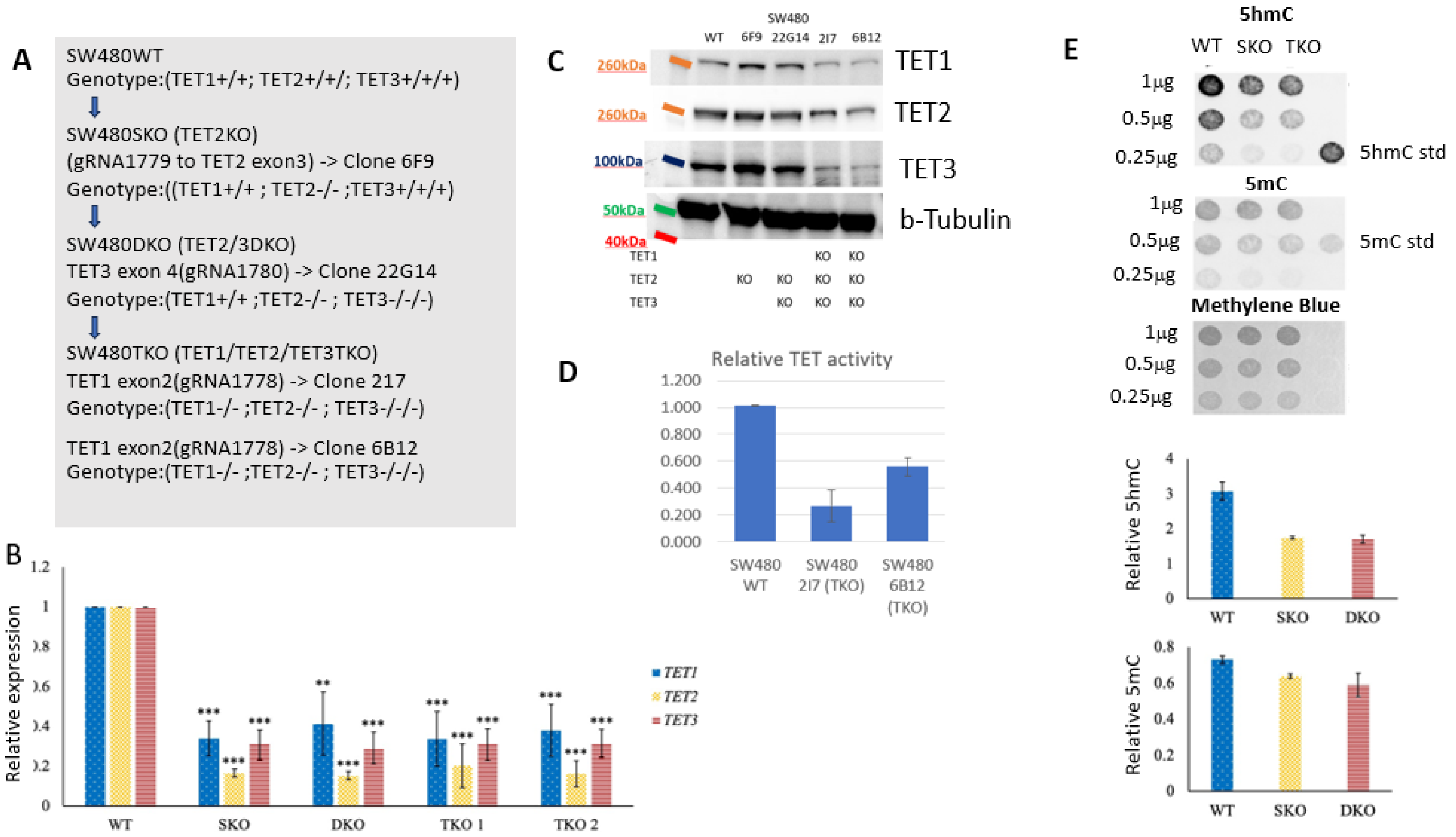

Figure S4 CRISPR-Cas9 strategy for TET1/TET2/TET3 triple knock out (TKO) in SW480 cells and effects on 5hmC accumulation. A) Schematic of knockdown strategy; B) Effect of knockdown in SW480 cells on *TET* transcript levels as measured by PCR. SKO is TET2KO, DKO is TET2/TET3KO, The error bars represent the standard deviation of 3 independent replicates. P> 0.01 (one-way Anova, followed by Tukey single-step multiple comparison); C) Western blot analysis to determine TET protein levels after knockdown; D) TET activity in knockout cells relative to wild type measured by colorimetric 5mC hydrolase TET assay kit (ABCAM). Two biological replicates (two sets of nuclear extracts per cell line) and three technical replicate assays for each sample. Activity normalised relative to wild type. E) Representative Immunoblot analyses of 5hmC and 5mC in triple knockout (TKO) and wildtype SW480 cells, with a bar chart below showing quantitative immunoblot confirmation of reduced 5hmC in the two TKO clones. Error bars show standard deviation of four independent replicates. Supporting data values are in Additional File 6.
